# Addictive behaviors among healthcare workers: A bicentric Tunisian Study

**DOI:** 10.1192/j.eurpsy.2023.348

**Published:** 2023-07-19

**Authors:** A. Omrane, H. Dabbebi, I. Mlouki, T. Khalfallah, S. EL Mhamdi

**Affiliations:** ^1^ Occupational Medicine and Ergonomics; ^2^Preventive and Community Medicine, Faculty of Medicine of Monastir Tunisia, Monastir, Tunisia

## Abstract

**Introduction:**

Addiction is a disease that comes with a range of consequences. Its physical, mental, personal, social and financial repercussions could not be neglected. Yet, this issue is still stigmatized. It is a public health problem that may affect people from different backgrounds including healthcare professionals.

**Objectives:**

To investigate the risk factors for a substance addiction: cigarette addiction and behavioral addiction: internet addiction among health care professionals.

**Methods:**

A cross-sectional bi-centric study was conducted among healthcare workers from two public hospitals in the center-east of Tunisia. A questionnaire was distributed to those who gave their oral consent to take part in the study. It included items related to participants’ socio-demographic characteristics, medical history, self-esteem and mood assessment. It combined questions from the Rosemberg self-esteem scale (RSES) and the Beck Depression Inventory alongside with validated tests: Adverse Childhood Experiences (ACE-IQ), the Fagerstrom Test for Cigarette Dependence and the Internet Addiction Test (IAT).

**Results:**

Among respondents, 16.1% were smokers. Risk factors for smoking were: being a male (OR=9.62), being in contact with patients (OR=4.75), a job tenure exceeding ten years (OR=3.11), having regular alcohol consumption (OR=7.27), unprotected sex (OR=9.24), depression (OR=3.87) or having suffered from sexual abuse during childhood (3.07). Nevertheless, practicing sport regularly (OR=0.32) and anxiety (OR=0.23) were identified protective factors. A high level of cigarette addiction was observed with 34.3% of cigarette smokers. Predictors of high cigarette dependence were: job tenure over ten years (OR=20.69), regular alcohol consumption (OR=6.11) and unprotected sex (OR=7.14). Among healthcare workers, 70.8% reported a normal internet use. Internet addiction was specially seen with those who were not engaged (OR=2,92) and those who worried about being unsuccessful (OR=1.91). Good self-esteem and being older were protective factors with OR=0.49 and OR=0.38 successively. Depression did not contribute to the development of internet addiction, on the opposite it protected against it (OR=0.36).

**Conclusions:**

Cigarette and internet addiction are threatening health problems that need more effort to screen and address. This public health issue has never stopped growing with the emergence of new types of illicit drugs and behaviours. Finally, this study draws attention to the importance of assessing the prevalence of different categories of addiction in Tunisia and highlights the necessity of updating policies used to address these addictions in a timely and appropriate way.

**Disclosure of Interest:**

None Declared

